# Hypnosis-associated blue-tinted vision: a case report

**DOI:** 10.1186/1471-2415-5-28

**Published:** 2005-12-01

**Authors:** Ran D Anbar, Aaron D Savedoff

**Affiliations:** 1 Department of Pediatrics, University Hospital, State University of New York Upstate Medical University, Syracuse, NY, USA

## Abstract

**Background:**

Self-hypnosis has been taught routinely at the SUNY Upstate Medical University for treatment of pulmonary symptoms thought to be amenable to psychological therapy. While using hypnosis for relaxation, four individuals, including a patient with cystic fibrosis, reported development of blue-tinted vision. Based on a search of the literature, we believe this is the first published report of hypnosis-associated blue-tinted vision.

**Case presentation:**

The patient reported blue-tinted vision when he used hypnosis on an almost daily basis for seven years. The visual change typically occurred when he was relaxed. Moreover, a concurrent erection in the absence of sexual thoughts usually was present. The other three individuals reported blue-tinted vision after learning how to use hypnosis for relaxation as part of a group hypnosis instruction.

**Conclusion:**

The blue-tinted vision experienced by the individuals in this report may be the result of an hypnosis-induced primary change in cognitive processing. Additionally, as the relaxing effect of hypnosis can be associated with a reduction in blood pressure and increased blood flow, hypnosis-associated blue-tinted vision also may be related to retinal vasodilation.

## Background

Self-hypnosis has been taught routinely at the SUNY Upstate Medical University Pediatric Pulmonary Center for treatment of pulmonary symptoms thought to be amenable to psychological therapy [1]. Patients who have benefited from hypnosis instruction include those with asthma, cystic fibrosis (CF), habit cough, shortness of breath, and vocal cord dysfunction [1,2].

Our understanding of the nature of hypnosis is contested, and rapidly evolving [1]. In practice, hypnosis can be defined as an altered state of consciousness during which a therapeutic goal can be achieved as a result of suggestions made by a therapist or self-suggestions given by a patient [1]. The state of hypnosis is a natural mental state that can be achieved, for example, by children engaged in imaginary play, or when students become bored and imagine themselves engaged in an activity elsewhere.

After being taught to use hypnosis for relaxation by a physician from our Center, four individuals, including a patient with CF, reported development of blue-tinted vision. Based on a search of the literature, we believe this is the first published report of hypnosis-associated blue-tinted vision.

## Case presentation

The patient was a 15-year-old with mild CF who was non-adherent to his prescribed therapies. As a result of this behavior, he developed shortness of breath and abdominal discomfort. His prescribed medications included pancreatic enzymes, a multivitamin, an antacid, long and short-acting bronchodilators by metered dose inhalers, a corticosteroid by metered dose inhaler, and a mucolytic medication by nebulization. The patient was supposed to undergo daily mechanical chest physiotherapy. In order to maintain his weight, he was supposed to eat a diet consisting of at least 3500 kcal/day.

The patient was the second of six children. His youngest sister had CF as well, while the other siblings were healthy. He lived with both parents. When he was asked why he had difficulties adhering to his therapies, he stated that he was frustrated by his family's poor financial situation and insufficient supervision for the children.

On physical examination, he appeared small for his stated age, but in no distress. His height was 149 cm (below the 3^rd ^percentile for age, and at the 50^th ^percentile for a 12-year-old), and his weight was 35 kg (below the 3^rd ^percentile for age, and at the 50^th ^percentile for an 11-year-old). His head, eyes, ears, nose, and throat examination was normal. His lungs were clear to auscultation. There was no murmur on cardiac examination. His abdominal examination revealed no organomegaly or tenderness. His pubic hair was at Tanner stage II. He had 3+ digital clubbing.

The patient wondered whether use of self-hypnosis might help him become more adherent to his therapies, as he was aware that hypnosis was used widely by patients at his Cystic Fibrosis Center [3]. He was told that he could use hypnosis as a tool to become more adherent if that is what he wished. The patient did not undergo testing for hypnotizability.

He was taught two induction techniques as a demonstration of how imagery in the mind could affect his body. He learned how to imagine his hands as two giant magnets that attracted each other, and noted how his hands came together, apparently "on their own." He imagined holding a pail full of sand in one hand, while holding helium balloons in the other hand. Within 15 seconds, one hand fell slowly to his lap and the other hand levitated. The patient stated it felt as if his hands had moved of their own accord.

He then was instructed in how to use imagery in order to relax. He learned to imagine going to a relaxing place, which he picked as being in his bedroom. He imagined what he might have perceived with each of his senses there. He relaxed his muscles progressively from head to toe. He picked the triggering gesture of touching his right index finger to his right thumb that would remind him how to relax even when he is not in hypnosis. When he alerted, he stated that the hypnotic experience made him feel as if he was really in his bedroom. When he made his triggering gesture, he appeared visibly more relaxed and said he was very pleased with his hypnotic abilities.

The patient was asked to which of his therapies he wanted to adhere better. He replied that he wished he could remember to take his pancreatic enzymes because otherwise he might continue to develop abdominal discomfort. He said he loved food. Therefore, it was suggested that he imagine a shield in front of his mouth that would not move in order to permit him to eat, unless he "unlocked" the shield by taking his enzymes.

A month later, the patient reported taking his enzymes regularly. Further, his adherence to the other therapies also had improved. He had used self-hypnosis to rid himself of headaches by employing his triggering gesture. He asked how he might use hypnosis to help himself feel better. It was suggested that he pass a healing light through himself when he did hypnosis. He used this imagery, and when he alerted he reported noting a bluish tint to his vision.

For the subsequent seven years, and continuing at this time, the patient used self-hypnosis on an almost daily basis. He has utilized it to relax, resolve headaches and stomachaches, achieve complete analgesia in his extremities while undergoing medical procedures, reinforce his adherence to his therapies, and to plan his daily activities. Also, he used the hypnotic technique of automatic word processing [4] to express his feelings about the challenges of having a terminal disease. He developed a long-term successful sexual relationship with a girl during these years.

When queried about the blue tint of his vision, the patient reported that the phenomenon continued to occur approximately half of the time when he used hypnosis, typically when he was very relaxed. He reported that the visual change caused him to perceive imagery with a blue background while he was in hypnosis. The blue-tinted vision typically would persist for two to ten minutes following completion of an hypnosis session.

He said he had noted that whenever he developed blue-tinted vision he also had a concurrent penile erection. He said that he was surprised about the erection because he was not having sexual thoughts. Also, he stated that his erections were noticeable, because while the rest of his body became very relaxed, he became very aware of the sensations in his groin. Further, he reported that blue became lighter in color when his erection became stronger. He reported that on one occasion he remained in hypnosis for three hours. During this time his blue vision "came and went", as did his erection.

The patient stated that he was unaware of reports regarding the association of sildenafil and blue-tinted vision. Further, the physician who taught the patient how to use hypnosis, also was unaware of this association at the time the patient first reported development of blue-tinted vision.

The three other individuals who reported blue-tinted vision in association with hypnosis, were part of a California group of 38 relatives of patients with CF, including adolescents and adults, who did not know the patient because he lived in New York. All 38 relatives were instructed in use of hypnotic imagery to achieve relaxation, in the same manner as described for the patient. Following the 6-minute group hypnosis session, one of the women reported spontaneously that her vision appeared blue transiently following hypnosis. When the rest of the group was queried, another woman stated that she also had developed transient blue-tinted vision. A 17-year-old boy stated that he had developed blue-tinted vision in the past while doing hypnosis, which persisted for approximately 15 minutes following hypnosis. No group member reported any other color or visual changes in association with the hypnosis. Because of the group setting, no inquiry was made regarding any other bodily changes that might have been associated with the blue-tinted vision.

## Discussion

Studies have demonstrated that color perception in the brain can be altered by imagery and suggestion in highly hypnotizable subjects [5]. Other visual changes reported to be inducible by hypnotic suggestion include development of color blindness, tunnel vision, and visual hallucinations [6-10]. Further, it is thought that hypnosis can improve a subject's subjective feeling of visual acuity by improving other cognitive functions such as memory, attention and perceptual learning [10]. Thus, the blue-tinted vision experienced by the individuals in this report may be the result of an hypnosis-induced primary change in cognitive processing. Additionally, as visual changes were not suggested and persisted after completion of hypnosis, it is possible that the blue-tinted vision developed as a result of hypnosis-induced retinal vasodilation mediated through the autonomic nervous system [11-13]. Such vasodilation may occur as a result of the known association of hypnosis with a reduction in blood pressure and increased blood flow [11-13].

Blue-tinted vision as a result of hypnosis may be similar in nature to the blue-tinted vision that has been reported as a rare side effect of sildenafil, a phosphodiesterase (PDE) inhibitor [14]. Sildenafil has been demonstrated to induce retinal vasodilation, perhaps as a result of PDE5 inhibition [15]. Hypnosis-associated changes in color perception are unlikely to be related to visual disturbances associated with digitalis toxicity (including development of yellow vision) because the effect of digitalis is thought to be caused by its inhibition of sodium-potassium adenosine triphosphatase [16], which is unlikely to be affected by hypnosis.

Hypnosis can be used as part of treatment for erectile dysfunction, by relaxation training, and suggestions aimed at improvement of self-confidence [17,18]. While the patient in this report did not suffer from erectile dysfunction, it is possible that his erections occurred because of hypnosis-associated relaxation and improvement in self-confidence. Additionally, the erections may have occurred as a result of hypnosis-induced vasodilation of the arterioles associated with the corpus cavernosum. This vasodilation may be related to the effects of hypnosis on the balance between the sacral parasympathetic and sympathetic activity that is associated with the development of penile erection [19]. Such vasodilation may be analogous to that caused by sildenafil, which typically is used for treatment of erectile dysfunction [14].

Sildenafil is thought to cause penile erections as a result of vasodilation caused by PDE5 inhibition, while it is believed to cause blue-tinted vision as a result of minor inhibitory action against PDE6, an enzyme that resides exclusively in retinal rod and cone receptors [20]. Since sildenafil became available widely in 1998, other phosphodiesterase inhibitors have been introduced to treat erectile dysfunction such as vardenafil and tadalafil. Sildenafil is 9 × more selective at inhibiting PDE5 than PDE6, while vardenafil is 16 × more selective, and tadalafil is 1000 × more selective [14]. Altered color perception has not been reported with use of vardenafil and tadalafil, thereby supporting the idea that PDE6 inhibition leads to development of blue-tinted vision [14]. On the other hand, sildenafil has been available for widespread use for much longer than tadalafil or vardenafil. Therefore, it is possible that the rare side-effect of blue-tinted vision as a result of PDE5 induced vasodilation has not yet been observed with the newer phosphodiesterase inhibitors.

The described patient was successful at inducing vivid imagery such as imagining that his bedroom appeared "real". His ability to use a triggering gesture to return back to the relaxed state demonstrated a good acceptance of a post-hypnotic suggestion. Further, using hypnosis the patient was able to achieve complete analgesia in his extremities during medical procedures. Thus, he demonstrated several traits characteristic of highly hypnotizable children [21]. It is not known whether the other three individuals reporting blue-tinted vision could have been classified as highly hypnotizable. Therefore, it is unclear whether development of blue-tinted vision typically might be achieved only by individuals who are highly hypnotizable.

## Conclusion

The blue-tinted vision experienced by the individuals in this report represents an anecdotal finding. Controlled studies can help define whether blue-tinted vision occurs as a result of an hypnosis-induced primary change in cognitive processing or is related to retinal vasodilation, and its relationship with hypnotic responsiveness of the subjects. In order to gain a better understanding of this phenomenon, affected individuals might be studied as well through measures of retinal vasodilation.

## Competing interests

The authors declare that they have no competing interests.

## Authors' contributions

RA is the physician who treated the patient in this report for cystic fibrosis, provided the described hypnosis instruction, and revised the manuscript. AS performed the literature search for the report, wrote the initial draft of the manuscript, and participated in its revision.

## Pre-publication history

The pre-publication history for this paper can be accessed here:


